# Selenium-silk microgels as antifungal and antibacterial agents†

**DOI:** 10.1039/d3nh00385j

**Published:** 2024-01-18

**Authors:** Elizabeth G. Wiita, Zenon Toprakcioglu, Akhila K. Jayaram, Tuomas P. J. Knowles

**Affiliations:** a Centre for Misfolding Diseases, Yusuf Hamied Department of Chemistry, University of Cambridge Lenseld Road Cambridge CB2 1EW UK tpjk2@cam.ac.uk; b Cavendish Laboratory, Department of Physics, University of Cambridge J J Thomson Avenue Cambridge CB3 0HE UK

## Abstract

Antimicrobial resistance is a leading threat to global health. Alternative therapeutics to combat the rise in drug-resistant strains of bacteria and fungi are thus needed, but the development of new classes of small molecule therapeutics has remained challenging. Here, we explore an orthogonal approach and address this issue by synthesising micro-scale, protein colloidal particles that possess potent antimicrobial properties. We describe an approach for forming silk-based microgels that contain selenium nanoparticles embedded within the protein scaffold. We demonstrate that these materials have both antibacterial and antifungal properties while, crucially, also remaining highly biocompatible with mammalian cell lines. By combing the nanoparticles with silk, the protein microgel is able to fulfill two critical functions; it protects the mammalian cells from the cytotoxic effects of the bare nanoparticles, while simultaneously serving as a carrier for microbial eradication. Furthermore, since the antimicrobial activity originates from physical contact, bacteria and fungi are unlikely to develop resistance to our hybrid biomaterials, which remains a critical issue with current antibiotic and antifungal treatments. Therefore, taken together, these results provide the basis for innovative antimicrobial materials that can target drug-resistant microbial infections.

## Abstract 2

Silk-based microgels that contain selenium nanoparticles embedded within the protein scaffold, that display potent antibacterial and antifungal properties, while importantly remain highly biocompatible with mammalian cells.

## Graphical abstract

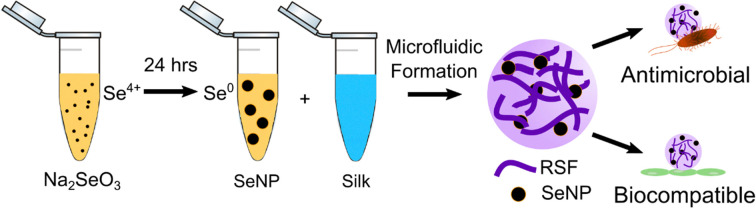

**New concepts**

We report the formation of a novel silk-based, micron-sized hydrogel (microgel), decorated with selenium nanoparticles, that displays potent antimicrobial activity, while also being completely biocompatible. By employing the use of droplet-microfluidics, we systematically form microgels of uniform size, a key component for controlling antimicrobial activity. As therapies involving conventional metallic-based nanoparticles typically have limited biocompatibility with mammalian cells due to their cytotoxic effects, their use in biomedical applications has been limited. In this work, we overcome this obstacle by incorporating the selenium nanoparticles with a protein scaffold, which is able to protect the mammalian cells from the cytotoxic effects of the bare nanoparticles while simultaneously serving as a material for microbial eradication. This allows for the use of ultra-low selenium nanoparticle concentrations, that still have strong antimicrobial activity. To our knowledge, this is the first report describing the synthesis of silk microgels combined with selenium nanoparticles, which are capable of effectively eradicating Gram positive/negative bacteria and, importantly, also fungi. We believe that our approach can be further extended to other nanomaterial systems to form effective antimicrobials in the global fight against antimicrobial resistance.

## Introduction

Antimicrobial resistance (AMR) is a leading threat to global health, causing an estimated 4.95 million deaths in 2019, 1.27 million of which were due to bacterial AMR.^1,2^ Many factors have contributed to this issue including the misuse and overuse of antibiotics and antifungals.^3^ This has resulted in the emergence of multidrug-resistant strains of both pathogenic bacteria and fungi, also referred to as “superbugs,” which are not treatable with current therapeutic methods.^3,4^ Advances in detection of bacterial and fungal strains can help with AMR management and response; however, innovation is needed to help eradicate and prevent the spread of these microbes.^5^ Metal-based nanoparticles such as Ag, Cu, and Zn have been shown to effectively overcome drug-resistant mechanisms but require optimisation in order to limit cytotoxicity towards healthy tissue.^6–9^ Previously, it has been shown that integrating nanoparticles into biomaterials reduces the cytotoxicity of the nanoparticles while maintaining the antimicrobial activity.^10^ This discovery is critical as bare nanoparticles are known to be quite cytotoxic due, in part, to the presence of reactive oxygen species (ROS), which can cause various kinds of cellular damage, including cell membrane disruption, DNA damage, and ATP depletion.^11^ Additionally, by employing an element such as selenium, which is an essential micronutrient and trace element in the human body, the selenium nanoparticles are shown to be more biocompatible at low concentrations than other inorganic nanoparticles.^12,13^ To that effect, efforts have previously been taken to use selenium nanoparticles as a route for antimicrobial applications.^14–17^

Hydrogels derived from proteins and peptides are unique materials for biomedical applications due to their biocompatibility and biodegradability.^18–23^ These qualities allow them to be used effectively in soft tissue repair and as engineering scaffolds.^24–28^ One specific class of proteins that are highly suitable for such applications due to their biodegradability and lack of cellular toxicity is silk-derived proteins and, more specifically, regenerated silk fibroin (RSF).^29–32^ RSF has the propensity to self-assemble into fibrillar networks that possess strong mechanical properties similar to that of native silk.^33^ Importantly, silk offers several advantages over cotton when used as a biomaterial.^34^ Wound dressings aim to protect lesions from further injury, dehydration, and infection.^35–37^ Using silk as a wound dressing effectively addresses these needs as it is an excellent hydrophobic barrier, making it effective at preventing absorption of outside particles.^38^ Moreover, this property prevents silk from degrading in the same way that cotton does upon exposure to aqueous environments, thus making silk-derived proteins excellent candidates for numerous biomedical applications.

In this study, we demonstrate the synthesis of silk-based microgels decorated with selenium nanoparticles using a droplet microfluidic strategy. The resulting hybrid selenium-silk microgels were characterised using electron and confocal microscopy. Employing a microfluidics-based approach allowed us to control and finely tune the size and properties of the microgels. Furthermore, the antibacterial and antifungal activity of the formed microgels was investigated. We found that the microgels displayed potent antimicrobial activity against both Gram-negative, *E. coli*, and Gram-positive, *B. subtilis*, bacteria. Additionally, the microgels displayed strong antifungal properties against *C. parapsilosis*. We determined that the microgels eradicate bacteria by disrupting the bacterial membrane using a dye, SYTOX blue, which is only able to enter cell membranes that have been damaged. Importantly, the selenium-silk microgels are highly compatible with mammalian cell lines and did not exhibit any cytotoxicity. We determined that adding the silk protein to the selenium nanoparticles improved the biocompatibility when compared to that of the free selenium nanoparticles. This result suggests a protective mechanism in which the silk acts as a barrier for human cells and does not allow for the toxic effect of nanoparticles to inhibit cellular viability. The resulting hydrogel material, has the potential to be used with *in vitro* and *in vivo* applications. Most studies conducted thus far focus on using selenium nanoparticles as either an antibacterial or antifungal agent but not both. The work presented here targets both bacteria and fungi simultaneously and provides the foundation for a new approach to fabricating biomaterials specifically for combating antimicrobial resistance.

## Results and discussion

**Fig. 1:**
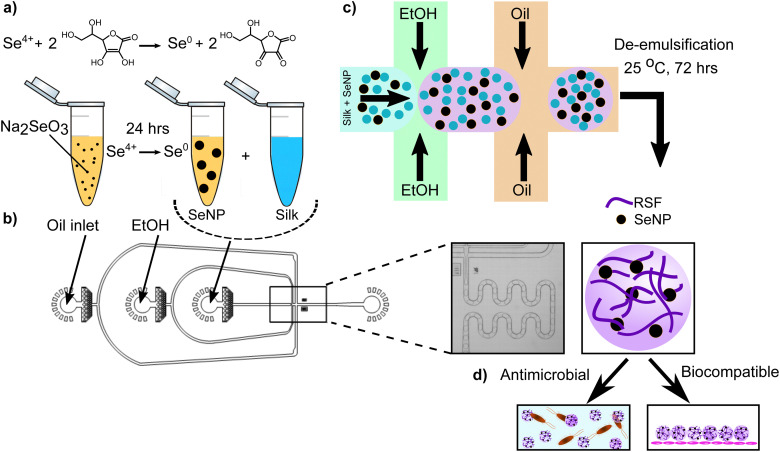
Schematic showing the synthesis of regenerated silk fibroin (RSF)-selenium microgels. (a) Redox reaction for the synthesis of selenium nanoparticles (SeNPs). Sodium selenite was first reduced by ascorbic acid and incubated for 24 hours in order to promote the synthesis of SeNPs. (b) The materials were loaded onto a microfluidic device containing two junctions. Oil was added into the top inlet, followed by ethanol, and finally by the SeNP-silk mixture. Microdroplet formation occurred when the oil phase intersected with the aqueous phase in the second junction. (c) Water-in-oil droplets were formed and kept at room temperature for 72 hours to promote protein self-assembly, before being de-emulsified, resulting in the formation of selenium-silk microgels. (d) The selenium-silk microgels are antimicrobial and biocompatible.

To develop a novel, antimicrobial therapeutic material, we utilised a microfluidics-based approach.^10,39^ First, we generated selenium nanoparticles (SeNPs) following a previously established protocol.^40^ In brief, sodium selenite was prepared at various concentrations. The selenium solution was then reduced using ascorbic acid at 56.7 mM and incubated at room temperature for one day to promote nanoparticle formation (Fig. 1a). We were able to control nanoparticle size by varying the sodium selenite concentration, with sizes ranging from roughly 60 to 140 nm.^41^ The ability to fine-tune nanoparticle size is of critical importance in biological applications as it can impact cellular interactions and the corresponding pharmacokinetics.^42,43^ The SeNPs were found to have uniform shapes and morphologies that make them easy to integrate into materials for delivery applications (ESI,† Fig. S1a and b). Moreover, we further characterised the SeNPs by conducting dynamic light scattering (DLS) and zeta potential measurements. These results corroborate that our nanoparticles are in fact on the order of 100–150 nm, while they have a negative charge of around −25 mV. These results are shown in ESI,† Fig. S2.

After synthesising the SeNPs, microgels were formed using a microfluidics-based approach wherein SeNPs and silk protein are initially mixed with ethanol before intersecting the oil phase on chip (Fig. 1b), resulting in the formation of micron-sized droplets. Silk protein was used due to its propensity to self-assemble and form fibrillar networks. Furthermore, the regenerated silk fibroin (RSF) used in this study is FDA approved, aiding the potential application of this system in a healthcare setting.^10,18,30,44–46^ Following microdroplet formation, monomeric RSF encapsulated within water-in-oil droplets self-assembled to form a nanofibrillar gel after incubating the microdroplets for 72 hours (Fig. 1c). This process is thermodynamically and kinetically favorable and occurs because the silk fibroin contains repeating hexapeptides GAGAGS and GAGAGY, which create hydrophobic groups in the chain that promote the formation of a β sheet structures and corresponding fibrils.^47,48^ The resulting product is a silk-based microgel with SeNPs dispersed throughout the spherical structure (Fig. 1d), where silk fibrils help to stabilise the SeNPs by preventing nanoparticle aggregation, as shown in (ESI,† Fig. S1a and b). The oil phase was then removed from the microgel emulsion using a previously established de-emulsification process.^10^ The de-emulsified microgels were re-immersed in water and imaged using brightfield microscopy, in order to observe whether the oil phase had been completely removed, which can be seen in ESI,† Fig. S3. The work presented here illustrates the basic concept for an antimicrobial material that has the potential to be used in wound healing applications by using biocompatible, protein-based, micron-sized hydrogels (Fig. 1d).

**Fig. 2:**
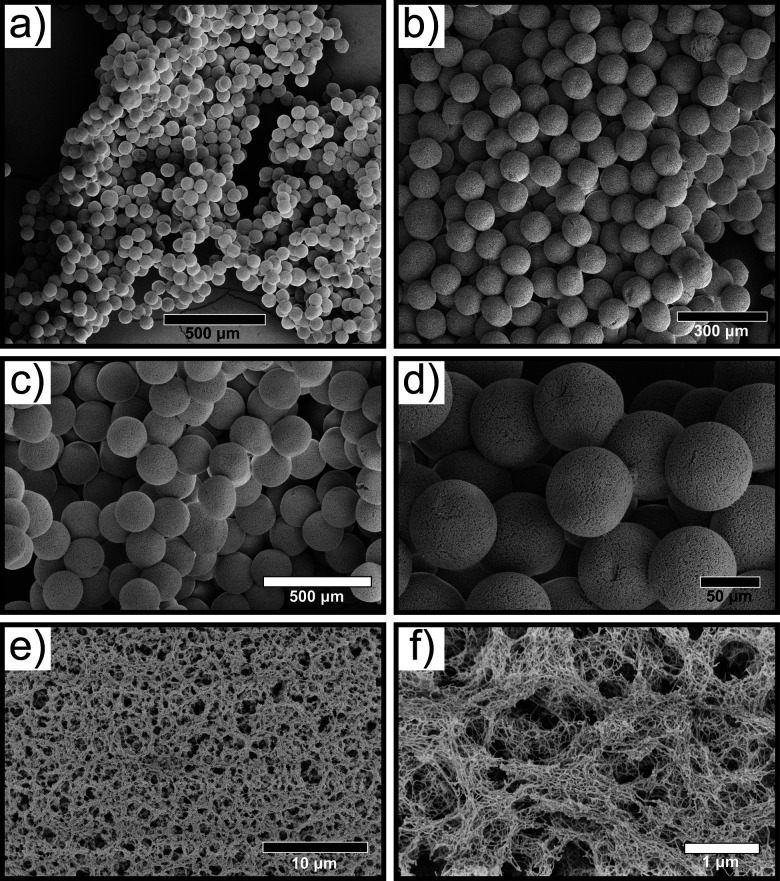
(a)–(d) SEM micrographs of selenium-silk microgels formed microfluidically. (e) and (f) High magnification micrographs depicting the dense silk fibrillar network within the microgel.

Following selenium-silk microgel formation, we then investigated their morphology and structure using microscopy. Scanning electron microscopy (SEM) was employed to characterise the selenium microgels and RSF nanofibrils. Selenium microgels were imaged by first using critical point drying (CPD) on the sample and then using high magnification SEM to take corresponding micrographs. The microgels were found to have a spherical and uniform morphology (Fig. 2a–d). This confirmed their high degree of homogeneity which is unique to the microfluidic method of fabrication. Furthermore, the dense fibrillar network, as shown in Fig. 2e and f, illustrates that the RSF nanofibrils are weaved throughout the whole microgel structure. From the SEM micrographs, the particle diameters were measured and were found to be approximately 70 μm in diameter. This size was further confirmed using confocal and brightfield microscopy, as seen in ESI,† Fig. S1c, d and S3. These results indicate that even when the microgel is dried, the size remains stable. Additionally, we investigated the stability of the microgels over time. To that effect, we monitored their stability for over 12 months and did not observed any degradation. Furthermore, this result is in agreement with other studies, where the stability of microgels was monitored over time and similar results were obtained.^10^ Brightfield microscopy images of microgels that were formed more than a year ago illustrate that they do in fact remain stable over months if not years (ESI,† Fig. S4).

In order to study whether nanoparticles are potentially released from the microgels, we conducted a release study (ESI,† Fig. S5). Two selenium microgel concentrations were prepared, 250 μg mL^−1^ and 50 μg mL^−1^. The nanoparticle release was monitored for a period of two weeks. It was determined that very small amounts of SeNPs were released from the microgels over the course of two weeks. After incubating the selenium microgels in water, a cumulative release of 0.029% and 0.034% for the 50 μg mL^−1^ and 250 μg mL^−1^ respectively, was detected after two weeks. Furthermore, it appears the release plateaus after 5 days of incubation. The microgels were intentionally designed to release minimal amounts of nanoparticles because bare nanoparticles are known to be exhibit high levels of toxicity to mammalian cells.^11,49^

**Fig. 3:**
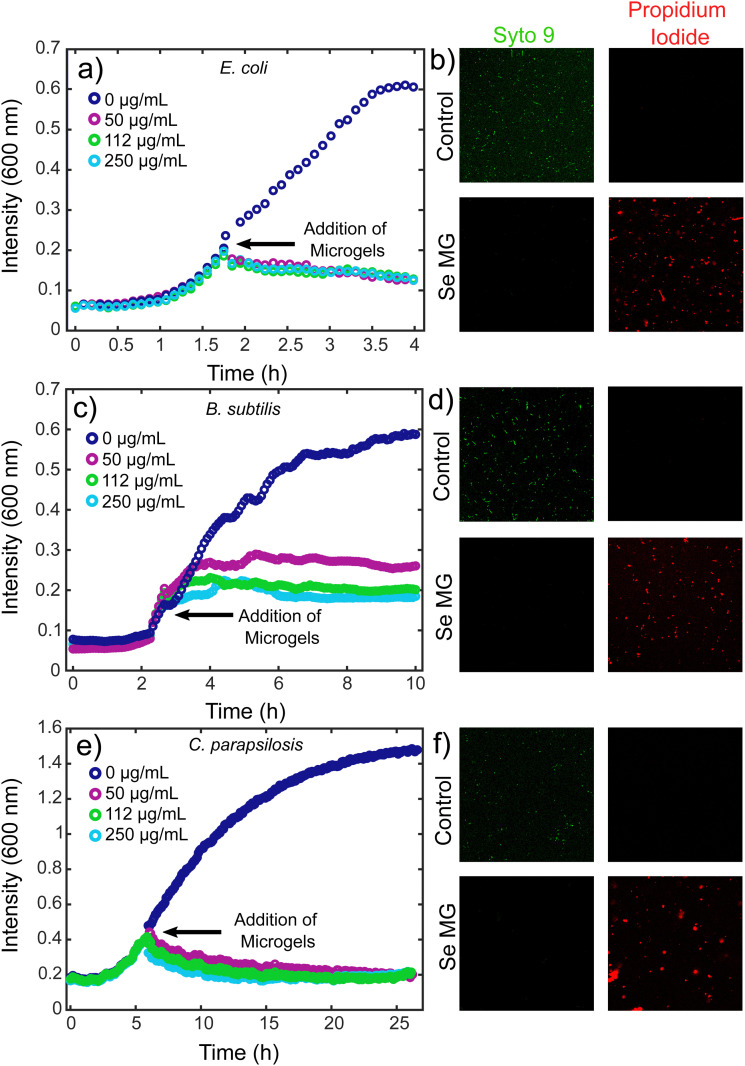
Kinetics of bacterial and fungal growth inhibition *via* turbidity analysis. The absorbance measurements were conducted at 600 nm for (a) *E. coli*, (c) *B. subtilis*, and (e) *C. parapsilosis*. Bacterial viability analysis was conducted using confocal microscopy with live/dead staining. Syto 9 (green) indicates live cells while propidium iodide (red) indicates dead cells. The (b) *E. coli* controls are shown in top two panels and indicate live cells. The *E. coli* with selenium-silk microgels are shown in lower two panels and indicate dead cells. Similar layouts are shown for (d) *B. subtilis* and (f) *C. parapsilosis* where bacterial and fungal death respectively was only observed in the presence of selenium-silk microgels.

In order to assess the use of these microgels as antimicrobial agents, we looked at both bacteria and fungi using *E. coli*, *B. subtilis*, and *C. parapsilosis*. The antimicrobial activity of the selenium microgels was tested using a kinetic growth analysis along with a live/dead assay, which involed the use of confocal microscopy. In order to evaluate the ability of the microgels to inhibit microbial growth at a level similar to that of an active infection, our microgels were tested with higher microbial load samples at a mid-log phase. The Gram-negative bacterium, *E. coli* and Gram-positive bacterium, *B. subtilis* were grown in a 96 well plate until a mid-log phase was reached, at which point, selenium microgels were added to the bacterial solutions. The concentrations of selenium-silk microgels we tested were 50 μg mL^−1^, 112 μg mL^−1^, and 250 μg mL^−1^. Complete inhibition of growth was observed upon the addition of microgels for all selenium concentrations tested for *E. coli* (Fig. 3a), while a slight concentration dependence was seen for the *B. subtilis* bacteria. After a marginal increase in bacterial growth, eventual stabilisation was reached that resulted in complete bacterial growth inhibition (Fig. 3c). Similarly, the antifungal activity of *C. parapsilosis* was investigated in the same manner. *C. parapsilosis* was added to a 96-well plate and allowed to grow until the exponential growth stage. Selenium-silk microgels were then added to the wells and fungal growth activity was monitored over time. Again, complete fungal growth inhibition was observed for the selenium concentrations tested (Fig. 3e). This fungal inhibition is of critical importance given the often overlooked rise in antifungal resistance.^50^ Lower concentrations of selenium-silk microgels were tested for the *E. coli* and *C. parapsilosis*, which can be found in ESI,† Fig. S6a and b respectively. We found that going to a slightly lower selenium concentration of 45 μg mL^−1^ did not result in strong microbial inhibition, suggesting that we have reached a potent antimicrobial limit at 50 μg mL^−1^ of selenium-silk microgels. However, upon addition of more selenium-silk microgels, even after the bacteria were allowed to proliferate, total bacterial eradication was observed, suggesting that bacterial resistance to our hybrid material is unlikely to occur.

Bacterial and fungal viabilities were also assessed using Syto 9 (green) and propidium iodide (red), which indicate live and dead cells respectively. *E. coli* was grown to an OD of 0.2 and the dyes were added to the bacteria. The control *E. coli* cells appeared green, indicating no bacterial death, as was expected (Fig. 3b top panels). However, when selenium-silk microgels were added to the *E. coli*, bacterial death was observed (Fig. 3b bottom panels). Similar results were found for the Gram-positive bacteria *B. subtilis*. When *B. subtilis* without selenium-silk microgels was imaged using confocal microscopy, only live bacteria were found (Fig. 3d top panels). Upon addition of selenium-silk microgels, bacterial death was observed (Fig. 3d bottom panels). Furthermore, antifungal activity was investigated using *C. parapsilosis*. The fungi without selenium-silk microgels remained alive (Fig. 3f top panels), while fungal death was observed (Fig. 3f bottom panels) when selenium-silk microgels were added to the solution. These results confirm the findings of the kinetic growth assays.

**Fig. 4:**
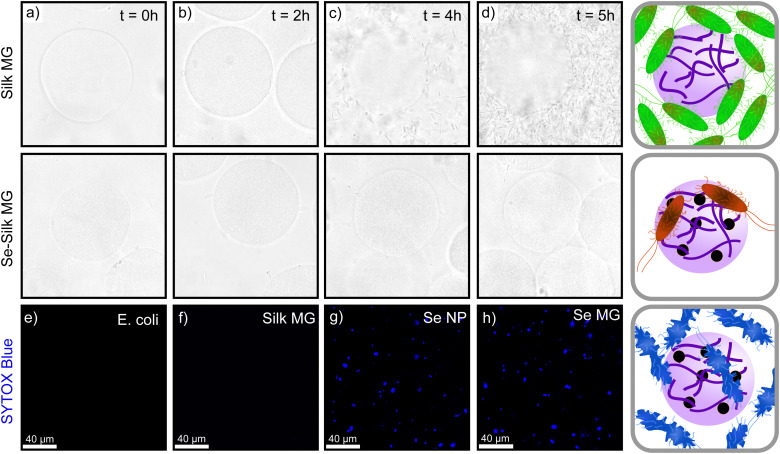
Mechanism of microbial eradication. (a)–(d) Bacterial activity was monitored with *E. coli* on a brightfield microscope. Top row illustrates that the silk microgels alone do not inhibit bacterial growth, while the bottom row shows that the selenium-silk microgels eradicate the bacteria. (e)–(h) Confocal images with SYTOX Blue cell stain. (e) *E. coli* control and (f) *E. coli* incubated with silk microgels do not fluoresce, indicating that the cell membranes are intact. (g) *E. coli* incubated with bare SeNPs and (h) *E. coli* incubated with selenium-silk microgels fluoresce, indicating disruption of bacterial membranes.

In order to understand the role of selenium in microbial death, we formed plain silk microgels that did not contain selenium. Bacteria were grown until the kinetic growth phase was reached and then silk microgels were added to the bacteria. The silk microgels were shown to have no effect on the growth of bacteria,^10^ indicating that the selenium is specifically responsible for microbial death. The same result was obtained and is shown in (Fig. 4), where bacteria grows on silk microgels alone but not with selenium-silk microgels. This result confirms our findings that selenium nanoparticles act as an antimicrobial source. Additionally, in order to understand the mechanism of microbial eradication, a comparative study between plain silk microgels and selenium-silk microgels was undertaken. Selenium-silk microgels and plain silk microgels were formed and separately incubated with *E. coli* and monitored over time. Confocal and brightfield microscopy was employed to image the microgel and *E. coli* mixture over a 5 hour time period. The selenium-silk microgels (Fig. 4 bottom row), have minimal bacteria present at *t* = 0 h. By 5 h, there is no visible increase in bacteria present with the selenium-silk microgels (Fig. 4d). Without the presence of selenium, bacteria proliferate and grow overtime (Fig. 4d). This result strongly suggests that the silk acts as a nutrient source for the bacteria and attracts the bacteria when they are in a solution together.^51^

To gather further mechanistic insights into the microgel mode of antimicrobial action, a membrane permeation assay using SYTOX Blue was performed. The cationic dye, SYTOX Blue, is unable to enter a healthy, intact cell. It can only enter cells if the membrane has been disrupted, by binding to the intracellular nucleic acid material. By employing fluorescent microscopy and exciting the sample at 405 nm, we can visualize the cellular membrane disruption.^52^ The samples showed no membrane disruption in the control samples of just *E. coli* (as seen in Fig. 4e). Additionally, upon adding silk microgels to the *E. coli*, no fluorescent signal was observed, indicating that silk does not promote changes within the membrane structure (Fig. 4f). However, upon addition of selenium nanoparticles and selenium microgels separately to solutions of *E. coli*, high fluorescent signal was observed, indicating that the cellular membrane has been impacted (Fig. 4g and h). This disruption likely came from reactive oxygen species (ROS), which are known to bind to intracellular components, thereby disrupting the membrane.^11,53^ The mechanism by which SeNPs eradicate bacteria and fungi has been shown to be *via* the generation of ROS. The presence of ROS promotes oxidation of key bacterial extracellular components leading to damage to biological membranes.^11,53,54^ Furthermore, ROS can inhibit ATP production and DNA replication, which prevents the cells' built-in antioxidant defence system from working and can lead to cell damage or cell death.^55,56^ Moreover, it is important to note that the SeNPs continue to have antimicrobial capabilities when combined with silk to form a microgel as the diffusion of ROS is not significantly impeded by the gel network.

**Fig. 5:**
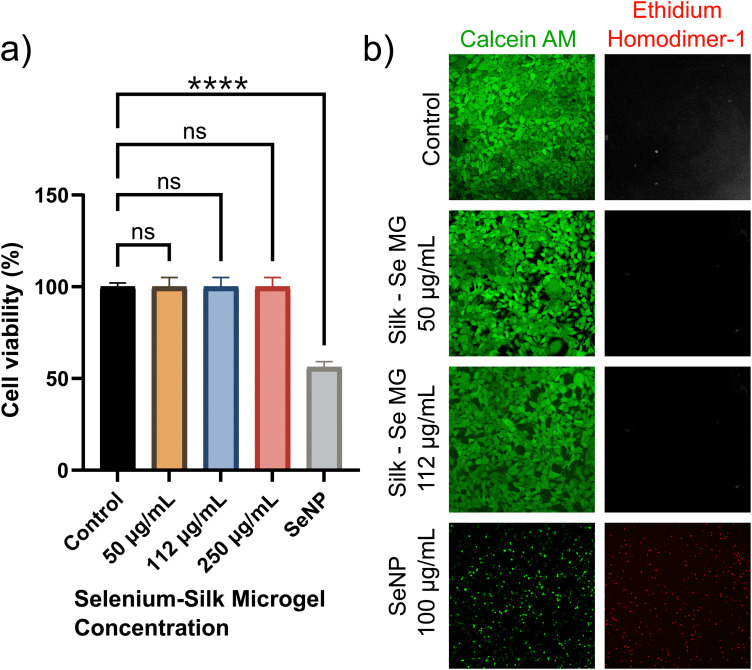
(a) Cytotoxicity analysis of selenium-silk microgels and bare SeNPs. A one-way ANOVA test was conducted, and in all cases, no significant difference in viability between the control and the different microgel samples was observed. However, bare SeNPs are clearly less biocompatible than the hybrid selenium-silk microgels. n.s. = not significant. ****p < 0.0001. (b) HEK-293 cell viability with selenium-silk microgels. Cells were stained with calcein AM and ethidium homodimer-1, indicating live and dead cells respectively. Last two rows show images of bare SeNPs incubated with HEK-293 cells. Cell death is observed, indicating that the selenium-silk microgel formation helps improve the biocompatibility.

In order to assess the use of selenium-silk microgels as an antimicrobial agent, we first had to ensure that the materials were biocompatible with mammalian cells. Biocompatibility assays with HEK-293 mammalian cells were thus performed. Selenium-silk microgels were incubated with mammalian cells over a period of 24 hours in a 96-well plate. It was seen that the microgels had no impact on cellular viability, as monitored by a standard MTT assay (ESI,† Fig. S7). At least 3 individual experiments were conducted. A one-way ANOVA test was performed and it was found that no significant difference in viability between the control and microgel samples was detected. Concurrently, a live/dead assay was performed which corroborated the results observed using the MTT assay. Calcein AM and ethidium homodimer-1 were the dyes used to stain the live and dead cells respectively. The resultant confocal images showed that the microgels did not impact both viability and proliferation, in stark comparison to samples containing only SeNPs (Fig. 5a and b). Thus, these two assays show that encapsulating SeNPs within a silk microgel greatly enhances their biocompatibility.

These findings represent a potential alternative therapeutic for bacterial and fungal infections. Importantly, selenium is notably less toxic than other inorganic nanoparticles, since it is a trace element in the human body and ranges in average dietary intake from 40 μg to 134 μg per day.^12^ The selenium-silk microgels presented in this study are at similarly low concentrations of 50 μg mL^−1^, 112 μg mL^−1^, and 250 μg mL^−1^. These low concentrations of selenium are safe for humans, which are further illustrated by our cell viability studies (Fig. 5). These concentrations are especially important since the selenium concentrations used in this study are within the average daily intakes of selenium for adults, therefore allowing for the potential use of these materials in future applications, possibly even involving clinical trials. Furthermore, the microbes are unlikely to develop resistance towards bacteria and fungi, which is a critical problem with current small molecule therapeutics. This result combined with the reduced toxicity makes the selenium-silk microgels ideal biomaterials to combat microbial infections.

Taken together, the antibacterial, antifungal, and biocompatibility results indicate that the optimum concentration of selenium-silk microgels is 50 μg mL^−1^. At this concentration, we see complete bacterial and fungal inhibition, while maintaining extremely high cellular biocompatibility. Furthermore, this concentration is less than the daily average selenium intake, suggesting that utilisation of these microgels in humans would be potentially safe.

## Conclusions

Antimicrobial resistance continues to represent one of the leading threats to global health. Alternative therapeutics are thus needed to combat this resistance. In this study we synthesized SeNPs and combined them with silk protein in a microfluidic chip to form micron-sized hydrogels (microgels). The resulting selenium-silk microgels were characterised using SEM, TEM, and confocal microscopy, which illustrated their high degree of monodispersity and fibrillar morphology. The antimicrobial activity was evaluated using two different bacteria and one fungi: *E. coli*, *B. subtilis*, and *C. parapsilosis* respectively. The addition of selenium-silk microgels to these microbes resulted in complete eradication that was confirmed using confocal microscopy and kinetic growth assays. Furthermore, the mechanism of microbial death was determined and it was found that bacteria proliferated over time in the presence of plain silk microgels but that bacteria did not grow in the presence of selenium-silk microgels. This result further confirms that the addition of selenium is specifically responsible for microbial death. The biocompatibility of these materials was also investigated using an MTT-based cell viability assay. After incubating the selenium-silk microgels with HEK-293 mammalian cells, the cells remained highly compatible, indicating excellent biocompatibility. This differs from that of the bare selenium nanoparticles, which exhibit poor biocompatibility, suggesting that the silk protein protects the cells from the cytotoxic effects of bare nanoparticles. Notably, selenium is less toxic than other inorganic nanoparticles due to selenium's trace presence in the human body, therefore it is an excellent candidate for potential further use. The low concentrations of selenium presented in this study of 50 μg mL^−1^ are less than the daily selenium intake in adults around 40 μg to 134 μg.^12^ The unique biocompatibility of the selenium-silk microgels and potent antibacterial and antifungal activity thus paves the way for using this approach in several healthcare treatments that target microbial eradication.

## Materials and methods

### Microfluidic device fabrication

To fabricate a master, a soft photolithographic process was utilised.^57,58^ In summary, a silicon wafer was placed under vacuum and 50 m photoresist (SU-8 3050, MicroChem) was spin-coated onto the material. The wafer was then soft baked at 95 °C for 3 min. Following the soft bake, the film mask was placed onto the wafer and was then exposed to UV light. This step induces polymerisation. Next, the wafer was baked a second time at 95 °C for 30 min. Lastly, the master was developed using a solution of propylene glycol methyl ether acetate (PGMEA, Sigma-Aldrich). Following the fabrication of the master, a device was prepared by adding an elastomer polydimethylsiloxane (PDMS) with a curing agent (Sylgard 184, DowCorning, Midland, MI) in a 10 : 1 ratio. The device was then placed under vacuum to remove any air bubbles for one hour, followed by an incubation at 65 °C for 3 hours. After curing, the PDMS was cut out of the master and inlets and outlets were formed by punching holes into the PDMS. Lastly, the prepared PDMS was bound to a glass slide using a plasma bonder (Diener Electronic, Ebhausen, Germany).

### Synthesis of selenium-silk microgels

The resulting device was used to make microdroplets. The device was placed under a microscope where a Mikrotron High Speed Camera was used to ensure droplet generation occurred. The flow rates within the microchannels were controlled using neMESYS syringe pumps (Cetoni). Three separate syringes were filled with (1) a fluorinated oil solution (Fluorinert FC-40) with 2% w/w surfactant (RAN biotechnologies), (2) a 40% ethanol solution, and (3) a 1 : 1 protein selenium nanoparticle (SeNP) solution. The resulting microgels were then de-emulsified. The collected droplets were left to sir for 48 h at room temperature to aid the protein self-assembly and microcapsule formation. The droplets were washed three times with 1 mL of FC-40 oil in order to remove the surfactant. The remaining oil was removed and then a 1 : 1 ratio of deionised water and 20% 1*H*,1*H*,2*H*,2*H*-perfluoro-octanol (PFO, Alfa Aesar) was added to the microgels. The samples were centrifuged for 2 min at 1000 rpm in order to separate the phases. The supernatant was collected and washed a total of three times.

### Characterisation of selenium-silk microgels

Scanning electron microscopy (SEM) was utilised using a critical point drying method. In brief, the selenium-silk microgels in aqueous solution were exchanged with an ethanol solvent. This was done in a sequential manner by first incubating the microgels overnight in a solution of 20% ethanol and 80% water. The following evening, the microgels were incubated in a solution of 40% ethanol and 60% water. This incubation continued until a 100% solution of microgels in ethanol was achieved. This step ensures that the microgels maintain their size and shape. Next, the microgels in ethanol were placed onto a silicon wafer. This wafer was then placed on a multipin mount and sputter coated with a 5 nm platinum layer. The resulting SEM micrographs were taken using a FEI Verios 460 at 1 kV.

### Kinetic growth measurements

*E. coli* and *B. subtilis* bacteria were grown at 37 °C until the exponential growth phase was reached in LB media. *C. parapsilosis* fungi was also grown at 30 °C in YM media until the exponential growth phase was reached. Selenium microgels were then added to the 96-well plate in a 1 : 4 selenium microgel to microbe ratio. The kinetic growth measurements were observed using a FLUOstar Omega microplate reader (BMG Lab tech). The experiment was repeated three times and the data presented in this paper is an average of these three measurements taken.

### Microbial cell viability analysis

The bacteria, *E. coli* and *Candida parapsilosis* were incubated at 37 °C until an OD600 of 0.2 was observed. The selenium-silk microgels were prepared at final selenium concentrations of 50, 112, and 250 μg mL^−1^. These were then added to the bacteria and left to incubate for 1 hour. Similarly, *Candida parapsilosis* fungi was grown until an OD600 of 0.2 was reached. The selenium microgels were then added in the same manner. After incubating the microbes, confocal images were taken using Syto 9 and propidium iodide (live/dead BacLight Bacterial Viability Kit, Thermo Fisher Scientific, TFS) as dyes to indicate live and dead cells. The dyes were mixed with the microbes in a 1 : 1 ratio. A 40× oil and 60× oil objective were used (Leica TCS SP8 inverted confocal microscope).

### Mechanism of microbial eradication

Two portions of the bacteria, *E. coli*, were incubated at 37 °C and grown to an OD600 of 0.05. The selenium-silk microgels were added to one portion and the plain silk microgels were added to the second portion. The solutions were imaged every hour on a confocal microscope with brightfield function using a 60× objective (Leica TCS SP8 inverted confocal microscope). Confocal Microscopy was further employed to study the disruption of the cell membrane. A solution of *E. coli* in LB media was grown to an OD600 of 0.1. Next, the solution was left to incubate for 30 min in a solution of 1 μM SYTOX Blue (Thermo Fischer Scientific) at 37 °C. The samples were then imaged using confocal microscopy LSM 510, excited at 405 nm (Leica TCS SP8).

### Cell culture of HEK-293 cells

The medium used for culturing human embryonic kidney 293 (HEK-293) cells consisted of advanced Dulbeccos modified Eagle medium (DMEM; Thermofisher Scientific, TFS) and 10% fetal bovine serum (Merck), supplemented by 50 U mL^−1^ penicillin and 50 μg mL^−1^ streptomycin (TFS), 1% (v/v) GlutaMax (TFS), and 50 μg mL^−1^ gentamicin (TFS). The cells were cultured in 25 cm^2^ flasks and passed when confluent.

### Cytotoxicity and cell proliferation using MTT Assay on HEK-293 cells

Cellular proliferation of HEK-293 cells upon treatment with selenium-silk microgels was measured using an MTT assay (TFS). Cells were seeded at a concentration of 10^5^ cells per well in a 96-well plate. To this, 50 μL of microgels was added per well and incubated for 24 hours under standard conditions of 37 °C and 5% CO_2_. The MTT assay was then performed as per the protocol provided by the manufacturer. A FLUOstar Omega microplate reader (BMG Labtech) was used to measure the absorbance purple solution resulting from the solubilisation of formazan crystals.

### Viability analysis of HEK-293 cells

A live/dead viability/cytotoxicity kit (Invitrogen) was used to assess the viability of HEK-293 cells following incubation with selenium-silk microgels. Cells were seeded at a similar density as described above for the MTT assay. Post seeding, microgels were added to the wells and incubated for 24 hours. Calcein AM and ethidium homodimer-1 were used to stain the live and dead cells respectively. The cells were imaged using a Leica TCS SP8 inverted confocal microscope.

### Release study

Hanging well inserts (Millipore, 0.4 μm pore size) were filled with 300 μL of selenium microgels (250 μg mL^−1^ and 50 μg mL^−1^). Three replicates of each sample were prepared. The well inserts were placed in 24-well cell culture plates that contained 1000 μL of distilled water in each well as a release media. The plate was placed in an incubator equilibrated at 37 °C. The entire release media was removed every 24 hours and replaced with 1000 μL of fresh prewarmed media. The released was analysed by UV/vis spectroscopy where the sample was measured at 265 nm using a BMG plate reader. The average concentration of NPs released was calculated using a standard calibration curve.

### DLS and zeta potential

The average particle size of the selenium nanoparticles was measured using dynamic light scattering (DLS). The samples were added to 12 mm square cuvettes (Malvern) and the spectra were recorded on a Malvern Zetasizer Nano instrument. The zeta potential of the selenium nanoparticles was also measured using the Malvern Zetasizer Nano instrument with a folded capillary zeta cell (Malvern).

## Conflicts of interest

There are no conflicts to declare.

## Supplementary Material

NH-009-D3NH00385J-s001
