# Supplementary material for: Selenium-silk microgels as antifungal and antibacterial agents
Source: Nanoscale Horiz. 2024 Jan 18;9(4):609–19. doi: 10.1039/d3nh00385j (PMC10962633; doi:10.1039/d3nh00385j)
Supplement: NH-009-D3NH00385J-s001 [file NH-009-D3NH00385J-s001.pdf]

Electronic Supplementary Material (ESI) for Nanoscale Horizons.
This journal is © The Royal Society of Chemistry 2024

Selenium-silk Microgels as Antifungal and Antibacterial Agents

Elizabeth G. Wiita,[†,‡] Zenon Toprakcioglu,[†,‡] Akhila K. Jayaram,[†,¶] and Tuomas P. J. Knowles*[,†,¶]

†*Centre for Misfolding Diseases, Yusuf Hamied Department of Chemistry, University of Cambridge, Lensfield Road, Cambridge CB2 1EW, UK*

‡*These authors contributed equally*

¶*Cavendish Laboratory, Department of Physics, University of Cambridge, J J Thomson Avenue, Cambridge CB3 0HE, UK*

E-mail: tpjk2@cam.ac.uk

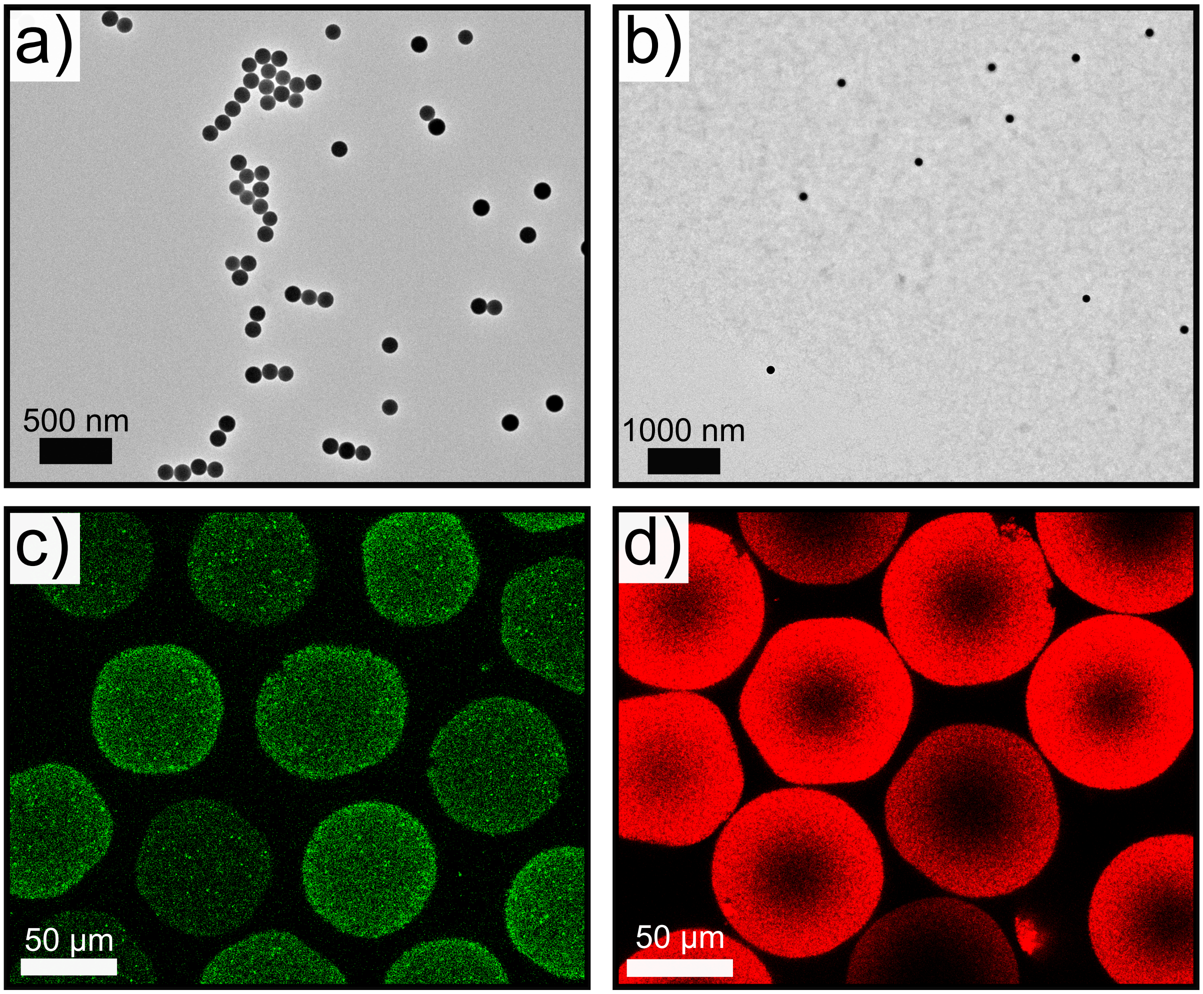

Figure 1: TEM micrographs for two samples: **(a)** 200 μg/mL SeNPs alone and **(b)** 200 μg/mL SeNPs with silk. SeNPs are dispersed throughout the silk fibrillar network. **(c-d)** Confocal images of selenium-silk microgels with Syto9 (green) and propidium iodide (red). Confocal images indicate that the microgels are approximately 70μm in diameter.

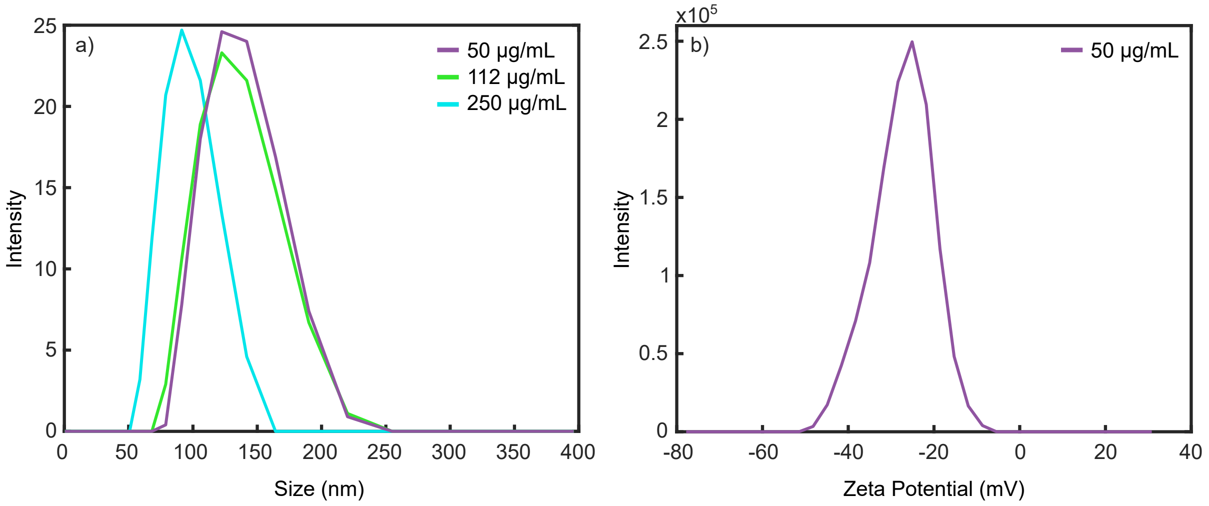

Figure 2: Dynamic light scattering data of the size distribution of SeNPs and the respective zeta potential values for the 50μg/mL.

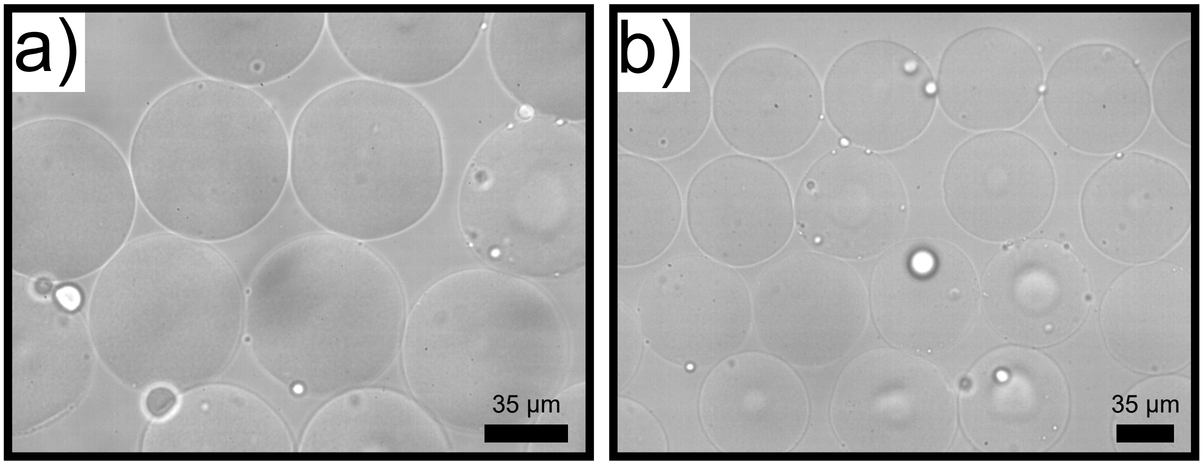

Figure 3: Optical microscopy images of de-emulsified selenium-silk microgels.

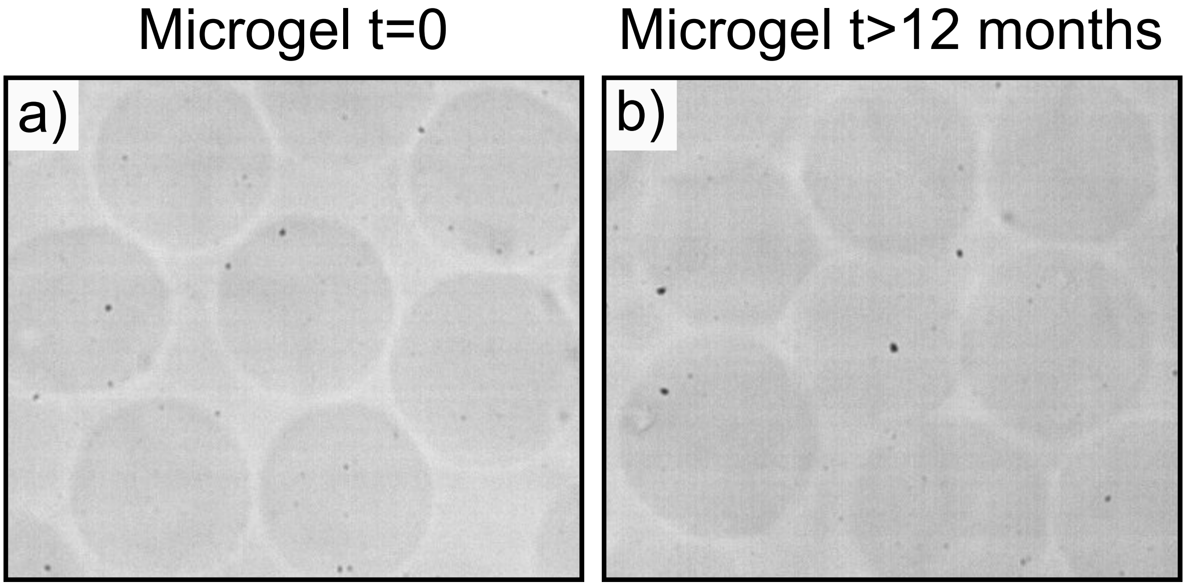

Figure 4: Optical microscopy images of microgels captured immediately following formation and after 12 months at room temperature.

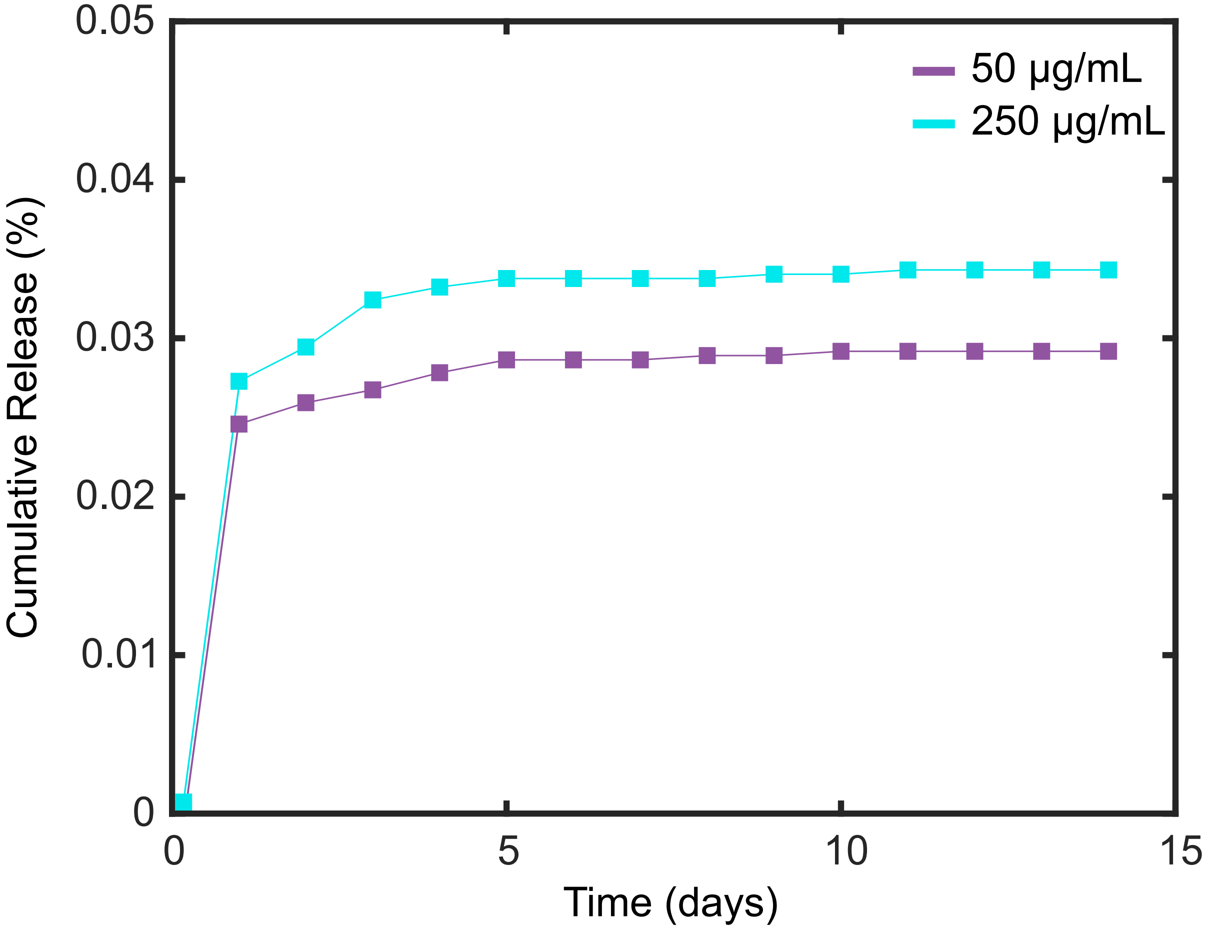

Figure 5: Cumulative release of nanoparticles from the microgels over a period of two weeks.

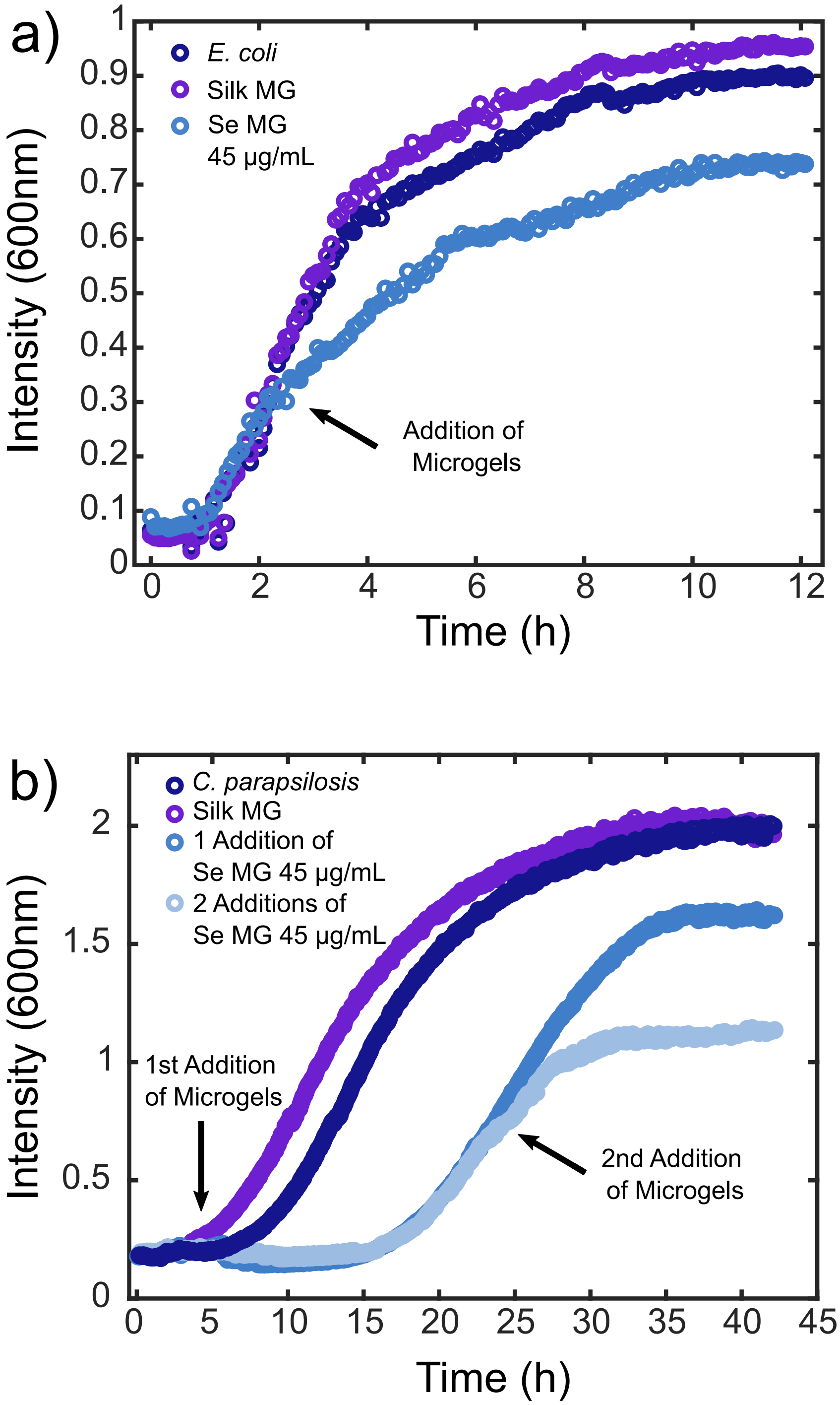

Figure 6: Kinetics of bacterial and fungal growth inhibition. Absorbance measurements were conducted at 600 nm for **(a)** *E. coli* **(b)** *C. parapsilosis*.

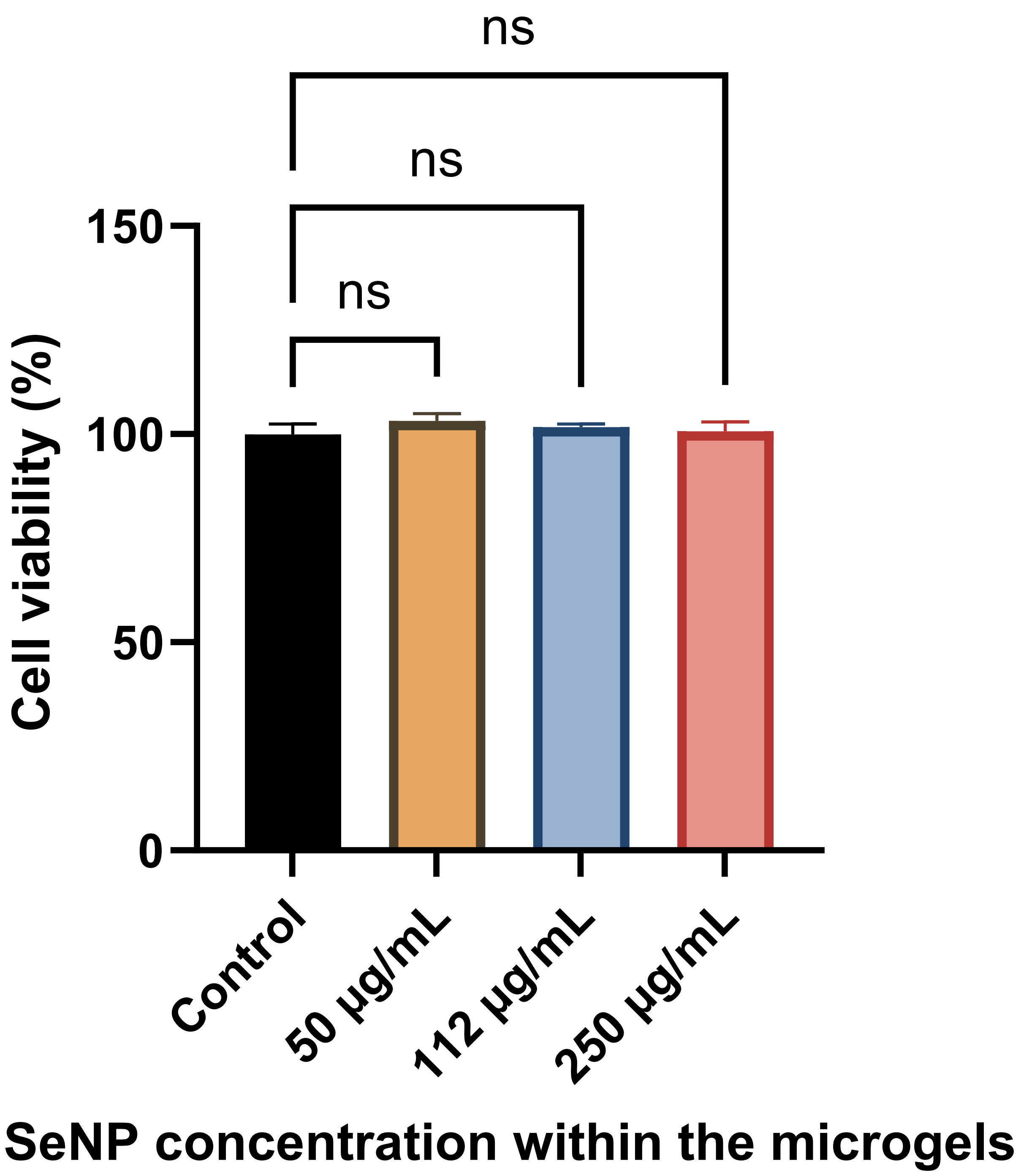

Figure 7: MTT assay of selenium-silk microgels. The data show the mean ± SEM of at least n = 3 individual experiments. A one-way ANOVA test was conducted, and in all cases, no significant difference in viability between the control and the different microgel samples was observed. n.s. = not significant.
